# Recent advances in melanin-like nanomaterials in biomedical applications: a mini review

**DOI:** 10.1186/s40824-019-0175-9

**Published:** 2019-12-03

**Authors:** Jihyo Park, Haeram Moon, Seonki Hong

**Affiliations:** Department of Emerging Materials Science, DGIST, Daegu, 42988 South Korea

**Keywords:** Melanin, Polydopamine, Bioimaging, Theranostics, Drug delivery, Biosensing

## Abstract

**Background:**

Melanins are a group of biopigments in microorganisms that generate a wide range of colorants. Due to their multifunctionality, including ultraviolet protection, radical scavenging, and photothermal conversion, in addition to their intrinsic biocompatibility, natural melanins and synthetic melanin-like nanomaterials have been suggested as novel nano-bio platforms in biomedical applications.

**Main body:**

Recent approaches in the synthesis of melanin-like nanomaterials and their biomedical applications have briefly been reviewed. Melanin-like nanomaterials have been suggested as endogenous chromophores for photoacoustic imaging and radical scavengers for the treatment of inflammatory diseases. The photothermal conversion ability of these materials under near-infrared irradiation allows hyperthermia-mediated cancer treatments, and their intrinsic fluorescence can be an indicator in biosensing applications. Furthermore, catechol-rich melanin and melanin-like nanomaterials possess a versatile affinity for various functional organic and inorganic additives, allowing the design of multifunctional hybrid nanomaterials that expand their range of applications in bioimaging, therapy, theranostics, and biosensing.

**Conclusion:**

Melanin-like natural and synthetic nanomaterials have emerged; however, the under-elucidated chemical structures of these materials are still a major obstacle to the construction of novel nanomaterials through bottom-up approaches and tuning the material properties at the molecular level. Further advancements in melanin-based medical applications can be achieved with the incorporation of next-generation chemical and molecular analytical tools.

## Introduction

Melanin refers to a group of biopigments in microorganisms, plants and animals that generates a wide range of colors from yellow to red (known as pheomelanin) and brown to black (called eumelanin). Melanin determines hair, eye, and skin color in humans and acts as a protective shield by adsorbing harmful ultraviolet (UV) light from solar radiation and scavenging reactive oxygen species (ROS) to prevent DNA damage to nearby cells [1]. Adsorbed solar energy can be dissipated as heat, contributing to the thermoregulation of animals, which is especially important for cold-blooded animals [2]. In the brain, neuromelanin is produced in some populations of catecholaminergic neurons with uncertain circumstances, with attention due to its hypothetical relationship to neurodegenerative disorders such as Parkinson’s disease [3, 4]. Melanin also plays a major role in the innate immune system of insects against bacterial and fungal pathogens [5]. Its multifunctionality and intrinsic biocompatibility have inspired scientists to develop novel nanomaterials by mimicking its chemical structure and material properties.

Among various precursors, dopamine (DA) has been a major precursor in the in vitro synthesis of melanin-like nanomaterials because it can be nonenzymatically crosslinked to 5,6-dihydroxyindole (DHI) under relatively moderate conditions, such as mildly basic pH (~ 8.5) and/or in the presence of dissolved oxygen in aqueous reaction solutions (details will be discussed in chapter 2.1). In addition to melanin-like properties, the versatile adhesive properties of DA-derived melanin (also called polydopamine, pDA) has allowed limitless applications in biomedical and environmental fields since it was first reported in 2007 [6]. pDA was first synthesized as a material-independent surface coating material that mimics the chemical composition of mussel adhesive proteins, and it has also been suggested as a synthetic model of natural melanin, in particular neuromelanin in the brain, to elucidate the unexplored mechanisms of natural melanin in nature [7]. pDA possesses various functional groups, such as protonated and/or unprotonated amine groups, catechols, and dihydroxyindole rings, that participate in multimodal molecular interactions on the surface of various types of materials constructing multifunctional coatings and adsorbing a bunch of molecules and ions as adsorbents (details will be discussed in chapter 2.2).

Here, we briefly review recent advances in natural melanin and melanin-like nanomaterials, such as pDA, that have been established for biomedical applications. Their synthesis and fabrication into nanoparticles, hollow nano/microcapsules, and core@shell structures with a controlled size and shape will be summarized in chapter 2, and their emerging applications in bioimaging, theranostics, sensors, and drug delivery systems will be introduced in chapter 3.

## Synthesis and characterization of melanin-based multifunctional nanomaterials

### Synthesis of artificial melanin

Melanogenesis in nature is initiated through the activation of tyrosinase, laccase and other polyphenol oxidases in response to external stimuli such as chemical stresses (metal ions, ROS, oxidizing agents), UV exposure, and pathogenic attack (particularly in insects) [8]. These enzymes convert the precursors (tyrosine, DOPA, and DA) into major intermediates, DHI and/or 5,6-dihydroxyindole-2-carboxylic acid (DHICA), that can be further polymerized and assembled into melanin granules. Inspired by biosynthesis in nature, the synthesis of melanin-like nanomaterials has been achieved with natural precursors as well as their synthetic analogs crosslinked via enzymes in vitro. F. Li et al. reported that pDA nanoparticles synthesized by laccase were more stable in strongly acidic and alkaline solutions than the chemically synthesized nanoparticles [9]. A. Lampel et al. systematically demonstrated the relationship between the structural variation of precursors and the resulting melanin-like material properties by tuning the amino acid sequence of self-assembled tripeptide crystalline precursors containing phenylalanine, aspartic acid, and tyrosine residues [10]. Enzymatic polymerization via tyrosinase not only formed covalent crosslinking between tyrosine residues but also altered noncovalent interactions in preordered crystalline tripeptides, resulting in a wide range of disordered melanin-like pigments synthesized. Surface-immobilized enzymes have allowed for the site-specific deposition of melanin-like nanoparticles in proximity to enzymes; however, the synthesis was self-terminated due to the uncontrolled adhesion of the synthesized particles to enzymes [11].

Chemical oxidation processes can be the ultimate alternatives to the enzymatic synthesis of melanin-like nanomaterials that allows for cost-effective mass production with reproducibility. Melanin-like pDA nanoparticles have been synthesized with tunable sizes ranging from a few nanometers to hundreds of nanometers in aqueous solution at mildly basic pH values by the addition of different ratios of sodium hydroxide (NaOH) or ammonium hydroxide (NH_4_OH) to DA. During these processes, X. Wang reported that radicals could further control the growth of the particles by either quenching the radical intermediates of pDA or accelerating seed formation [12]. Particle size was also affected by buffers; N. F. D. Vecchia et al. reported that the particle size of pDA increased with increasing initial DA concentration in phosphate or bicarbonate buffers, but inhibition of particle growth was observed in Tris buffer [13]. The authors suggested that the covalent attachment of Tris molecules to the surface of the growing pDA surface prevented particle growth. X. Jiang et al. confirmed the effect of the water-alcohol co-solvent system on pDA synthesis via an ammonia solution and established an empirical formula that predicts the size of the resulting particles in mixed solvents [14]. Chemical oxidants such as KMnO_4_, NaIO_4_, and CuSO_4_, and organic bases such as piperidine, have also been used for the synthesis of melanin-like particles and coatings in acidic pH conditions or in organic solvents [15–17]. Finally, temperature is another factor that can control the size of the growing particles [18].

Supramolecular assembly through noncovalent interactions has been suggested to be critical in regulating the shape and properties of melanin-like nanomaterials in addition to covalent crosslinking [19]. Theoretical and experimental studies have suggested various types of noncovalent interactions, such as hydrogen bonding, van der Waals interactions, and π-π stacking between oligomeric intermediates [20]. In 2012, Hong et al. experimentally isolated a trimer composed of DA and DHI assembled through unknown noncovalent interactions, suggesting that the building blocks for noncovalent assembly could be sufficiently low as oligomers, not polymers [21]. They subsequently reported experimental evidence in 2018 that cation-π interactions can be the major driving force for the molecular assembly of oligomeric intermediates into granules [22]. In addition to experimental studies, computational methods suggested that large molecular weight oligomers are less likely to form, and tetramers can be notably more stable than the other forms, implying that the major building blocks for noncovalent assembly can be quite repetitive, although the resulting granules possess diversity in their material properties [23].

### Synthesis of melanin-like hybrid nanomaterials

During both the enzymatic and chemical syntheses of melanin-like nanomaterials, additives can be simultaneously incorporated via either covalent crosslinking to growing oligomers or physically entrapped in particles through noncovalent interactions to tune their material properties (as summarized in Table 1). For example, fluorescent pDA nanoparticles were obtained in the presence of glutathione (GSH) as an additive to a DA precursor with potential applications in biosensing [47]. Ferric ion-complexed pDA nanoparticles were synthesized in one-pot and further annealed at 650 °C for 3 h, resulting in carbonized pDA embedded with 3–5 nm sized Fe_3_O_4_ as an organic/inorganic hybrid nanocatalyst [48]. A recent study by D. Wang et al. showed that DA precursors participated in constructing a metal-organic framework (MOF) with Mn^2+^ and organic linkers and further polymerized as a part of a MOF crystal, showing great potential for photothermal therapy (PTT) [49]. Hong et al. reported that cations such as the quaternary ammonium cation and potassium ions showed a remarkable affinity to building blocks of pDA via cation-π interactions, generating superhydrophilicity in one-pot synthesized coatings [22]. Targeting moieties as well as therapeutic agents could also be incorporated into growing melanin-like nanoparticles, with potential in bioimaging and theranostics [50] (details are shown in chapter 3.3).

**Table 1:** A summary of melanin-like nanomaterials synthesized for biomedical applications

Biomedical applications		Template	Size	Functional additives	Ref
Therapy	ROS scavenger	pDA NP	80 nm	–	[24]
			160 nm	w/ and w/o Cu^2+^	[25]
	Photodynamic therapy	pDA/PEG coating on CaCO_3_	40 nm	Photosensitizer: chlorin e6	[26]
	Photothermal therapy	pDA NP	160 nm	–	[27]
		PEGylated pDA NP	78 nm	Anticancer drug: doxorubicin, 7-ethyl-10-hydroxycamptothecin	[28]
	Drug delivery system	pDA NP	75–400 nm	Anticancer drug: camptothecin	[29]
		PEGylated pDA NP	28 nm	Ligand: triphenylphosphonium Anticancer drug: doxorubicin	[30]
		Polyethylenimine-decorated pDA NP	60–80 nm	Therapeutics: plasmid DNA	[31]
		Poly (methacrylic acid) -coated pDA capsule	300 nm	Anticancer drug: doxorubicin	[32]
		pDA coating on insulin NP	50, 71, 84 nm (coating)	–	[33]
		pDA/PEG coating on mesoporous silica NP	193.08 nm	Ligand: folic acidAnticancer drug: doxorubicin	[34]
Bioimaging and theranostics	Photoacoustic Imaging	Ethylenediamine/PEG-coated pDA NP	130 nm	–	[35]
		pDA-coated AuNP	150 nm	–	[36]
		PDA-coated Fe_3_O_4_ NP	15 nm	^64^Cu	[37]
		Melanin NP	42.5 ± 2.1 nm	Targeting moiety: poly-L-lysine	[38]
	Magnetic resonance imaging	pDA NP	51, 119, 188 nm	Cu (II)	[39]
		PEGylated pDA NP	70–300 nm	Mn (II)	[40]
			25–43 nm	Fe (III)	[41]
		PEGylated Melanin NP	13 nm	Gd (III), Cu (II)	[42]
Biosensing	Fluorescent pDA	pDA with ethylenediamine NP	Tens of nm	–	[43]
		pDA NP reduced by NaBH_4_	34.83 nm	–	[44]
	Fluorescence quencher	Mesoporous pDA NP	70 nm	Fluorescein amidites (FAM)-labeled DNA probe	[45]
		pDA-coated upconversion NP	5 nm (coating)	Cy3-aptamer	[46]

As-prepared melanin-like nanoparticles can be chemically reactive core templates for multifunctional core@shell nanostructures. Metal ions can chelate to the surface of nanoparticles for bioimaging, such as in photoacoustic imaging (PAI) [42] and magnetic resonance imaging (MRI) [40, 41]. Silver ions could be adsorbed and simultaneously reduced on the surface of artificial melanin, allowing for the green synthesis of reductant-free organic/inorganic hybrid nanoparticles with antibacterial activity [51]. Nucleophiles such as thiols and amines are commonly used moieties in immobilizing organic molecules on the surface of as-prepared melanin-like nanoparticles without the additional chemical reagents [6]. Thiolated polyethylene glycol could be used for the surface PEGylation of melanin-like nanoparticles to enhance the biocompatibility and stability in vivo [28, 35]. Targeting moieties such as RGD, folic acid, and triphenylphosphonium (TPP) could be immobilized on the surface of nanoparticles with anticancer drugs such as doxorubicin (DOX) and camptothecin (CPT) for tumor-targeting therapeutic delivery [29, 52–55] (details are shown in chapter 3.2). Interestingly, tumor cell lysates adsorbed on pDA nanoparticles acted as an immune booster for vaccine development in colorectal cancer [56].

Melanin-like nanomaterials, and in particular pDA, could be coated on the surface of any kind of template/core material to construct the reverse type of aforementioned core@shell nanostructures. pDA coating on the surface of inorganic nanoparticles enhances colloidal stability and prevents agglomerization and corrosion of inorganic nanoparticles [36, 37, 57–59]. In addition, it has been reported that pDA coating is not only biocompatible but also attenuates the intrinsic toxicity of inorganic materials such as quantum dots in vivo [60]. Melanin-like coating can also provide an adhesive nano-thin layer between the core and outer materials, expanding further design of theranostic nanomaterials with multifunctionality [61–64]. Moreover, by further modification of these processes, hollow melanin nanocapsules could be synthesized by dissolving sacrificial core materials of as-prepared core@melanin hybrid nanoparticles [26].

### Biocompatibility and biostability of melanin-like multifunctional nanomaterials

As a great candidate for biomedical applications, one of the strengths of melanin-like nanomaterials is their biocompatibility and long-term stability. They are not only well-dispersed in aqueous buffered medium, but also remained stable when stored in those solution without notable physical changes for up to at least several weeks [27, 59]. Coating of them to inorganic nanoparticles improve the water dispersibility and colloidal stability without altering thickness of the modification for long period of time [59], allowing a variety of both in vitro and in vivo biomedical applications.

Dopamine causes oxidative stress to cells and is cytotoxic, but it has been experimentally proven that melanin-like nanomaterials like pDA possesses good biocompatibility. In vitro cytotoxicity has been evaluated in many different types of mammalian cell lines including HeLa, 4 T1, HepG2, and NIH3T3; the cell viability was retained greater than 90% after incubating with those nanoparticles for up to several days [27, 35, 59, 65]. Hong et al. reported that there is still non-negligible amount of free dopamine that physically entrapped in synthesized pDA, but it is rarely released from pDA due to strong non-covalent interactions and therefore does not cause severe cytotoxicity [21]. In vivo studies have confirmed that the biocompatibility of those nanomaterials contribute to attenuate the intrinsic cytotoxicity of conventional biomaterials. Hong et al. reported that pDA coating significantly reduced the leukocyte population alternation caused by conventional CdSe core and ZnS-capped nanocrystals in blood after tail vein injection to mice [60]. In addition, it was reported to suppress the macrophage adhesion after 4 days and the foreign body giant cells (FBGCs) formation after 14 days from subcutaneous implantation of biodegradable poly (L-lactic acid) film in rats [60]. Similarly, Liu et al. investigated the long-term biocompatibility as well as ultra-stability of pDA coating in vivo; pDA-coated gold nanoparticles were mainly uptaken by the Kupffer cells in the liver, the organ with the highest accumulation, while they were uptaken by a variety of cells in the spleen, the organ with the second highest accumulation. They were intact within those cells for at least six weeks without causing notable histological toxicity in mice [59].

## Biomedical applications

### Anti-inflammatory and photothermal therapy

Melanin-like nanoparticles, and in particular pDA, have been suggested as therapeutics in the treatment of anti-inflammatory diseases because of the abundant phenolic groups that serve as radical scavengers. Zhao et al. demonstrated that 80 nm-sized pDA nanoparticles efficiently scavenged either H_2_O_2_^−^ or lipopolysaccharide (LPS)-induced cellular ROS in vitro, and further study in murine acute peritonitis models confirmed the successful suppression of in vivo inflammation [24]. Similarly, Bao and his colleagues validated the antioxidant capacity of 160 nm-sized pDA nanoparticles and provided potential applications in periodontal disease treatment to relieve oxidative stress without any side effects [25].

Abundant phenolic groups in melanin-like nanoparticles not only scavenge radical species but also quench photosensitizers such as chlorin e6 (Ce6). Photosensitizers generate ROS, including singlet oxygen species, to kill nearby cells upon light exposure in photodynamic therapy (PDT). The current drawback of conventional photosensitizers is their undesirable photoactivation [24]. To overcome this, Han and his colleagues developed complexed nanoparticles consisting of pDA and a photosensitizer conjugated to hyaluronic acid [66]. Since the synthesized nanoparticles release the photosensitizer from the quencher pDA only in response to the degradation of hyaluronic acid by the tumor-localized intracellular enzymes (e.g., hyaluronidase), tumor-specific photoactivation is allowed [66]. Similarly, Z. Dong et al. reported a calcium carbonate-pDA composite hollow nanoparticle with the loaded photosensitizer Ce6 that is released from pDA and photoactivated only in an acidic tumor environment [26].

Natural melanin and melanin-like synthetic nanoparticles possess broadband adsorption from the UV to near-infrared (NIR) range [67]. Therefore, they can provide efficient photothermal conversion upon NIR irradiation, which can be used in hyperthermia-mediated cancer treatments, also called PTT. The major advantage of PTT is the spatial control of light exposure for target-specific treatment, but the cytotoxicity of currently used photosensitizers and the limited penetration of the light source into the skin are obstacles in the widening of its application [68]. Recent studies have suggested that melanin-like nanoparticles can be the ultimate photosensitizers used in PTT because of their biocompatibility and biodegradability in addition to their strong NIR-reactive photothermal conversion efficiency. Liu et al. reported that melanin-like nanoparticles showed a photothermal conversion efficiency of 40% upon NIR irradiation with an 808 nm laser and did not cause long-term toxicity in rats in the absence of light exposure [27]. Furthermore, the synergistic effects of PTT and chemotherapy could be achieved by synthesizing drug-loaded pDA nanoparticles: the acidic nature of the tumor environment triggers the release of anticancer drugs such as DOX and 7-ethyl-10-hydroxycamptothecin (SN38), and the simultaneous hypothermia caused by light-excited pDA could successfully suppress tumor growth in a tumor xenograft model [28].

### Drug delivery

Currently, nanotechnology for drug delivery has received great attention because it allows for personalized healthcare by improving the targeting ability and drug loading efficiency to improve the therapeutic efficacy and reduce the side effects [69]. Therefore, a variety of nano-carriers with different sizes, structures and surface properties have been designed for smart drug delivery to date [69]. Among them, researchers have witnessed that melanin-like nanoparticles have many advantages in terms of their drug loading efficiency due to the strong π-π interactions between the aromatic rings on drugs and melanin in addition to their biocompatibility and biodegradability [70]. Furthermore, a variety of molecules could be physically adsorbed or chemically immobilized on artificial melanin to achieve multifunctionality. Table 1 summarizes the use of melanin-like nanomaterials in smart drug delivery systems.

Melanin-like nanomaterials can act as a core template, a coating or an adhesive layer between the core and the outer materials. Ho and his colleague compared the drug-loading efficiency and release profile of a model anti-cancer drug, CPT, adsorbed on the surface of as-prepared pDA nanoparticles in a size ranging from 75 nm to 400 nm, adjusted synthetically by pH [29]. As the size of the particles that were synthesized at a pH of approximately 7.5–8 increased, the drug loading efficiency also increased due to an advanced interior volume for the drugs of up to 11.81 μg per 1 mg of particles. In contrast to the loading efficiency, however, the drug release was much faster from the smaller particles, which were synthesized at pH 9 [29]. Cui and his colleagues developed a pH-dependent drug releasing platform by the synthesis of pDA hollow capsules decorated with DOX via a pH-cleavable linker [32]. Over 85% of the DOX showed a sustained released up to 12 h at pH 5, mimicking the tumor environment, whereas only 20% of DOX were released at neutral pH [32]. Targeting moieties could also be incorporated on the surface of artificial melanin templates with therapeutics for targeted drug delivery. W.-Q. Li et al. co-immobilized TPP with DOX on the surface of nanoparticles as the targeting moiety for mitochondria [30]. The developed nanoparticles can overcome drug resistance in long-term chemotherapy [30]. In addition, a gene delivery system was established by immobilization of amine-rich polymers such as polyethylenimine (PEI) on the surface of templated pDA NPs followed by complexation with plasmid DNA [31]. In this system, the toxic nature of PEI is suppressed by the conversion of the primary amine groups to secondary and tertiary amines during covalent conjugation with pDA [31].

Artificial melanin can also be an outer coating of nanomaterials to control drug release profiles and enhance biocompatibility and stability. A nano-thin coating layer of pDA on as-prepared insulin particles enabled pH-responsive insulin release [33]. Sustained release of insulin was obtained up to 40 h at pH 7.4, while only 29% was released at pH 5.4 [33]. W. Cheng et al. also employed pDA coating on DOX-loaded mesoporous silica nanoparticles as a pH-sensitive gatekeeper [34]. They further immobilized folic acid on the surface-coated pDA for targeted cancer therapy [34]. S. Li et al. reported that pDA coating with casein could stabilize the zein-resveratrol nanocomplex against environmental stresses, including pH, salinity, storage, redispersion, and UV irradiation, and enhance the antioxidant activity [71]. Finally, core-shell nanostructures have been synthesized by using artificial melanin-like pDA as an adhesive layer. M. Oroujeni et al. adopted pDA to immobilize the drug carrier 6-thio-β-cyclodextrin on the surface of magnetic nanoparticles, and hydrophobic diclofenac (DCF) was subsequently inserted into the surface-immobilized 6-thio-β-cyclodextrin [64]. Oligonucleotide-based aptamers and molecular probes have also been covalently immobilized on pDA-coated multifunctional nanoparticles via Michael addition reactions for potential applications in biosensors and nanomedicine [62].

### Bioimaging and theranostics

Photoacoustic (PA) imaging is a noninvasive, nonionizing, and low-cost imaging method based on converting irradiated light energy absorbed by target molecules into thermal energy [28, 36]. Melanin and its derivatives, including pDA, have been suggested as endogenous chromophores that can produce PA signals in vivo [35]. Zhang et al. synthesized a melanin-loaded nano-liposome for PA imaging- and MR imaging-guided photothermal cancer therapy, and the longitudinal relaxivity, *r*_*1*_, of the synthesized nano-liposomes was 0.25 mM^− 1^ s^− 1^ at 7 T [72]. Ju et al. reported that pH-induced aggregation of melanin nanoparticles resulted in increased PA signal strength by generating overlapping thermal fields between aggregated nanoparticles [35]. In addition, various metal ions and reduced metals, targeting moieties, and therapeutic agents are allowed to be integrated with melanin-like nanoparticles for multimodal imaging and theranostics. Cationic poly-L-lysine-coated melanin nanoparticles could be used for anionic glycosaminoglycans (GAGs)-targeted PA imaging for the early diagnosis of articular cartilage degeneration in osteoarthritis [38]. Repenko et al. reported that melanin-coated gold nanomaterials with various shapes could be used as gastrointestinal imaging probes for PA imaging with excellent dispersibility, minimal cytotoxicity, and augmented PA responses [36].

In addition to PA imaging, organic/inorganic hybrid nanomaterials composed of melanin-like nanomaterials and inorganic metal ions have emerged for MR imaging-based theranostics. High loading efficiency of various metal ions on melanin-like nanomaterials can be achieved for improved contrast, and further incorporation of poly (ethylene glycol) (PEG) increases the circulation time in vivo. Ju and his colleagues reported that the longitudinal relaxivity, *r*_*1*_, of Fe^3+^-loaded pDA nanoparticles was 17 mM^− 1^ s^− 1^ at 3 T, whereas that of the commercially available Gd-DOTA under the same conditions was 7.1 mM^− 1^ s^− 1^ [73]. Similarly, Gd^3+^-loaded 13 nm sized natural melanin was synthesized for MRI with high stability in vivo and a high *r*_*1*_ value [42]. Various metal ions, such as Fe^3+^, Cu^2+^ and Mn^2+^, could be loaded onto pDA nanoparticles for not only bioimaging but also simultaneous photothermal cancer therapy [39–41]; further co-immobilization of photosensitizers and therapeutic agents allows for multimodal theranostic applications. Wang et al. synthesized PEGylated-polydopamine (PDA) nanoparticles with the photosensitizer IR820 in addition to Fe^3+^ for MR imaging-guided combined photothermal and PDT [74]. pDA nanoparticles loaded with ICG, DOX, and Mn^2+^ could be used for MR imaging-guided combined chemotherapy and PTT in vivo [55]. Lin and his colleagues designed novel melanin-coated Fe_3_O_4_ nanoparticles with surface-adsorbed ^64^Cu isotopes for multimodal imaging-guided cancer phototherapy [37]. The high brightness observed on T1-weighted images, the high affinity for metal ions, strong absorbance in the NIR region, and biocompatibility of natural melanin contributed to the MR imaging, PA imaging and photothermal imaging/therapy, and ^64^Cu was employed for additional PET imaging [37].

### Fluorescence-based biosensing

UV irradiation induces the autofluorescence of both natural melanin and synthetic melanin-like nanomaterials [75]. Therefore, the in-situ synthesis of these fluorescent nanomaterials could be an indicator in biosensing applications. Kong et al. developed a GSH sensing platform based on the MnO_2_-induced synthesis of fluorescent polydopamine nanoparticles (FPNPs) as an indicator in the absence of GSH, because GSH inhibits FPNP synthesis by reducing MnO_2_ to Mn^2+^ [76]. Similarly, they also detected ascorbic acid by using CoOOH as an oxidant to synthesize FPNPs instead of MnO_2_ because CoOOH is reduced to CO^2+^ in the presence of ascorbic acid and loses its activity to synthesize FPNPs [77].

Furthermore, chemical modification and/or partial degradation could enhance the intrinsic fluorescence intensity of as-prepared melanin-like nanoparticles. Gu and his colleagues synthesized tens of nanometer-sized FPNPs via ethylenediamine-induced partial degradation of as-prepared pDA nanoparticles [43]. The synthesized FPNPs were photostable and biocompatible, so they could be applied to the bioimaging of neuromast hair cells in the lateral line of zebrafish [43]. Similarly, Lin’s group obtained FPNPs by using hydroxyl radicals instead of ethylenediamine because hydroxyl radicals can induce the partial degradation of as-prepared pDA nanoparticles by introducing hydroxyl groups onto the particles while reducing the π-π interactions [78]. FPNPs were used for the detection of Fe^3+^ via electron-transfer-induced fluorescence quenching after the chelation of Fe^3+^ to the catechol groups of the FPNPs [78]. Interestingly, Yin et al. reported that further chemical reduction could enhance the fluorescence intensity of the as-prepared FPNPs; they first synthesized FPNPs by sodium periodate-induced oxidation and then treated the FPNPs with sodium borohydride for the chemical reduction that tunes the surface of the as-prepared FPNPs to become full of hydroxyl groups [44]. The quantum yield of these FPNPs was 5.1%, which is the highest rank among the reported FPNPs so far [44].

In addition to the intrinsic fluorescent properties of melanin-like nanomaterials, these nanomaterials can be applied in biosensing applications as fluorescence quenchers. Wang et al. synthesized mesoporous pDA nanoparticles and applied them as quenchers for fluorophore-conjugated single-strand DNA (ssDNA) probes to detect the downregulated let-7a and upregulated miRNA-21 in different types of cancer cells [45]. ssDNA probes were adsorbed on the surface of pDA nanoparticles through noncovalent bonds, such as π-π stacking and electrostatic attraction, and then released from the surface by complexation with their complementary miRNAs, resulting in the recovery of their fluorescence [45]. Interestingly, mesoporous pDA nanoparticles successfully protected surface-bound ssDNA probes from cleavage by DNase I, which was used to target miRNA recycling for signal amplification [45]. Ma’s group synthesized a novel upconversion@pDA core@shell nanoparticle with surface-complexed Cy3-labeled aptamer probes for the detection of cytochrome C (Cyt C) in living cells. The fluorescence of the probes was quenched at the surface of the nanoparticles and then recovered by simultaneous dissociation and binding to Cyt C in the cytosol immediately after cellular uptake [45]. In addition, the steady upconversion luminescent signals from the core of the synthesized nanoparticles served for not only intracellular imaging but also as an internal standard for the quantitative measurement of Cyt C from Cy3-labeled aptamers [46].

### UV shielding in dermatology

Melanin protects nearby cells in skin against sunlight by absorbing harmful ultraviolet (UV) rays and scavenging free radicals that cause DNA damage. Thus, synthetic melanin-like nanomaterials can be emerging as biocompatible sunscreens and next-generation therapeutics for people with melanin deficiency, such as albinism and vitiligo, which increases the risk of skin cancer [7]. Huang et al. demonstrated that melanin-like nanoparticles with a diameter of about 200 nm can be endocytosed, undergo perinuclear aggregation, and form a supranuclear cap in human epidermal keratinocytes, similar to natural melanosomes occurring in human skin [7]. Accumulated melanin-like nanoparticles reduced reactive oxygen species (ROS) and prevented DNA damage in cells under UV irradiation [7]. C. Wang et al. suggested polydopamine-encapsulated polymeric gels as bioinspired sunscreens with superior UV shielding properties, nonphototoxicity, and nonirritating nature [79]. Similarly, Y. Wang et al. compared the UV shielding properties of polydopamine nanoparticles with natural melanin granules encapsulated in polymer matrix, indicating that decrease in the size of nanoparticles lead to increased UV-shielding and visible light transparency properties with reduced light scattering [80]. However, most of synthetic melanin-like nanomaterials up to date are brown-to-black due to the broad range absorbance from UV to near-IR range, which is a major obstacle in cosmetic applications. In addition to UV, absorbance manipulation in visible range of those materials can be the next-step to be achieved.

## Conclusion

Melanin is a biopigments group in nature that possesses multifunctionality including ultraviolet protection, radical scavenging, and photothermal conversion. Its multifunctionality as well as intrinsic biocompatibility have inspired researchers to design novel melanin-like nanomaterials. Various enzymatic and chemical oxidation processes from natural precursors and their synthetic analogues have been reported, and additives can be easily incorporated in situ or form core-shell structures that expand the achievable scope of multifunctionality. Synthesized melanin-like nanomaterials have been emerging in various biomedical applications including therapy, bioimaging, and biosensing. In addition, the electrical conductivity and ion transport of melanin in nature have opened the very recent applications in soft bioelectronics. However, their bottom-up design/fabrication and tuning material properties at the molecular level are still limited because the chemical structure of these materials has not been fully elucidated thus far. Understanding the heterogeneity of the chemical structure and molecular weight distribution of oligomeric/polymeric building blocks and the driving force for nanoscale assembly via next-generation chemical and molecular-level analytical tools will be the next step forward to advance melanin-based biomedical applications in the future.

## Data Availability

Not applicable.

## Abbreviations

Ce6: Chlorin e6
CPT: Camptothecin
Cyt C: Cytochrome C
DA: Dopamine
DCF: Hydrophobic diclofenac
DHI: 5,6-dihydroxyindole
DHICA: 5,6-dihydroxyindole-2-carboxylic acid
DOX: Doxorubicin
FBGCs: Foreign body giant cells
FPNP: Fluorescent polydopamine nanoparticle
GAG: Glycosaminoglycans
GSH: Glutathione
LPS: Lipopolysaccharide
MOF: Metal-organic framework
MRI: Magnetic resonance imaging
NIR: Near-infrared
PA: Photoacoustic
PAI: Photoacoustic imaging
PDA: PEGylated-polydopamine
pDA: Polydopamine
PDT: Photodynamic therapy
PEG: Poly (ethylene glycol)
PEI: Polyethylenimine
PTT: Photothermal therapy
ROS: Reactive oxygen species
SN38: 7-ethyl-10-hydroxycamptothecin
ssDNA: Single-strand DNA
TPP: Triphenylphosphonium
UV: Ultraviolet
